# Development of a New Feline Combination Vaccine Including a Novel RNA Particle Vaccine Against Feline Leukemia Virus

**DOI:** 10.3390/vaccines14070625

**Published:** 2026-07-16

**Authors:** Willem Huisman, Aart Mommen, Stephanie Basten, Kim Driessen, Peter Pepels, Qi Cao, Hans Holtslag, Melissa Ann Bourgeois, Jacqueline Pearce

**Affiliations:** 1 MSD Animal Health, 5831 AN Boxmeer, The Netherlands; aart.mommen@msd.com (A.M.); stephanie.basten2@msd.com (S.B.); kim.driessen@msd.com (K.D.); peter.pepels@msd.com (P.P.); qi.cao2@msd.com (Q.C.); hans.holtslag@msd.com (H.H.); jacqui.pearce@msd.com (J.P.); 2 Merck Animal Health, Rahway, NJ 07065, USA; melissa.bourgeois@merck.com

**Keywords:** vaccines, feline, RNA replicon, immunity, duration, vaccine safety, virus shedding

## Abstract

Background/Objectives: We describe the rationale, design and evaluation of a novel feline combination vaccine (Nobivac^®^ NXT HCPChFeLV) incorporating RNA particle technology against feline leukemia virus (FeLV) alongside live attenuated components for feline herpesvirus-1 (FHV), feline calicivirus (FCV), feline panleukopenia (FPL) and *Chlamydia felis*. Efficacy and safety outcomes from laboratory and field studies are presented. Methods: Nobivac NXT HCPChFeLV is a 0.5 mL, non-adjuvanted, lyophilized vaccine containing live attenuated FHV, FCV, FPL, and *C. felis* strains, and an alphavirus replicon-derived RNA particle expressing FeLV envelope glycoprotein (gp85). The primary vaccination schedule involved subcutaneous vaccination at 8–9 weeks of age with a booster 4 weeks later. Onset-of-immunity studies were performed 1 week post primary vaccination. Duration-of-immunity studies evaluated challenge both at 1 year and 3 years after primary vaccination with a booster at 1 year (FHV, FCV, FeLV); FPL duration was assessed at 3 years after primary vaccination; *C. felis* duration was assessed at 1 year. Clinical signs of disease, survival, leukopenia (FPL) and pathogen excretion were assessed. Safety was assessed in laboratory overdose/repeat-dose studies and in a field study comparing concurrent (non-mixed) use with rabies vaccination vs a positive control regimen. Results: Vaccination significantly reduced clinical disease following FHV, FCV and *C. felis* challenge and reduced FHV-associated mortality. Vaccinated cats were protected from FPL clinical disease, mortality, leukopenia, and FPL excretion. Persistent FeLV antigenemia was significantly prevented after primary vaccination and in 1-year-duration-of-immunity studies and was reduced 3 years after revaccination. The excretion of FHV, FCV and *C. felis* was significantly reduced in vaccinates vs controls. No serious adverse events were observed in laboratory studies; field vaccination was well tolerated with no injection-site reactions reported. Conclusions: Nobivac NXT HCPChFeLV provides robust, long-lasting protection against multiple feline pathogens, reduced pathogen shedding and demonstrated a favorable safety profile, supporting its use in feline preventive healthcare.

## 1. Introduction

Vaccination remains a cornerstone of feline preventive healthcare, substantially reducing morbidity, mortality, and pathogen transmission associated with highly consequential infectious diseases. Indeed, international guidelines issued by bodies such as the World Small Animal Veterinary Association (WSAVA) and the Advisory Board on Cat Diseases (ABCD) stress the importance of vaccination in preserving the health of cats [1,2]. According to the WSAVA vaccination guidelines, feline herpesvirus-1 (FHV), feline calicivirus (FCV), and feline panleukopenia virus (FPL) are considered core vaccines for all cats. Feline leukemia virus (FeLV) is also considered core for cats less than a year of age where endemic, and for adult cats at risk due to lifestyle (outdoor access or living with cats with outdoor access). Rabies vaccination is likewise considered core in areas where the disease is endemic. Non-core vaccines recommended for at-risk cats include those for feline immunodeficiency virus (FIV), *C. felis*, and Bordetella bronchiseptica [2].

Despite these clear vaccination recommendations, vaccine-preventable diseases in cats remain widespread and have a significant impact on the health of cats throughout the world. One review of calicivirus found that prevalence was lowest in healthy household cats kept in small groups (2.5%), higher in larger groups (32%), and highly variable in colonies and shelters (up to 50–90%) [3]. Similar to calicivirus, a review of herpesvirus found the disease to be common and variable depending on cat population sizes and intermittent shedding, ranging from <1% to 20% or even higher [4]. Feline panleukopenia is associated with an age-related susceptibility and is most commonly found in unvaccinated and incompletely vaccinated kittens. Mortality for this disease can range from 20 to 80%, depending on the supportive care and health history of the affected animals [5]. In certain regions, the prevalence of progressive FeLV in healthy cats has declined in recent years and now ranges from 2.3 to 3.3% in North America and 0.7 to 15.6% in Europe, reaching up to 38% in sick cats; however, this decreasing trend appears to have stabilized in many areas [6,7]. Conversely, some parts of the world report higher rates, with progressive infection reaching up to 34.4% in healthy cat populations tested in Brazil [8,9]. To prevent resurgence of these diseases in regions with low prevalence and reduce the impact of cases in high-prevalence areas, ongoing vigilance and continued vaccination efforts are crucial.

A substantial proportion of cats remain unvaccinated. For example, surveys from the US, UK and Ireland indicate that only 57–62% of cats had seen a veterinarian in the preceding year [10,11,12]. In South America, one study estimated that fewer than 35% of owned pets are vaccinated in Argentina, while in Brazil only about 9% of cats receive annual veterinary visits and vaccination [13]. A global survey of cat owners from 14 countries around the world found that only 71% of cats had seen a veterinarian in the last 12 months and only 65% were previously vaccinated [14].

Based on the pervasiveness of disease combined with significant opportunities for vaccination, there is a clear need to develop novel vaccine solutions for cats. Concerns about current vaccines—including their adjuvants, slow onset and short duration of immunity, and 1 mL dose volumes—pose challenges for cat owners and veterinarians. Emerging technologies offer opportunities to create solutions that better meet evolving patient needs. At the same time, changing disease trends globally demand vaccines that can provide broader and more relevant protection. New vaccines may help to increase the number of cats vaccinated and improve overall feline health and disease control.

This article describes the scientific rationale, product design, and iterative development of a new feline vaccine, Nobivac^®^ NXT HCPChFeLV. This low-volume (0.5 mL), extended-duration, non-adjuvanted vaccine combines modified and avirulent live fractions with RNA particle technology. We present immunogenicity and safety data from laboratory studies and controlled field trials, and we detail the development of the FeLV fraction, which uses RNA particle technology—a novel platform first introduced in feline vaccines in North America in 2024 [15].

The RNA particle vaccine described in this paper employs an alphavirus-based replicon derived from the attenuated TC-83 strain of Venezuelan equine encephalitis virus (VEEV) to express the FeLV envelope glycoprotein (gp85). In this platform, the FeLV gp85 gene replaces the VEEV structural genes, yielding a self-amplifying RNA that expresses the gene of interest, i.e., FeLV gp85, once it enters cells. Deletion of the VEEV structural genes renders the RNA particles propagation-defective. Following vaccination, these RNA particles target and bind dendritic cells, delivering the replicon RNA into these cells. For a limited period of time, these replicons self-amplify intracellularly, directing translation and the production of FeLV envelope glycoproteins. These glycoproteins are subsequently processed and presented by the dendritic cells to induce both innate and adaptive immune responses, including virus-neutralizing antibodies and T cell responses, for a robust, balanced, and long-lasting immune response [16,17]. Importantly, these vaccines are non-adjuvanted, do not spread, cannot revert to virulence, require relatively low doses of antigen, and are produced in systems with reduced serum and without the use of antibiotics, preservatives, or thimerosal. This platform is the first innovation in vaccine technology for cats in over 20 years.

## 2. Materials and Methods

### 2.1. Vaccines and Solvents

Nobivac^®^ NXT HCPChFeLV (MSD Animal Health, Boxmeer, The Netherlands) is a lyophilized combination vaccine containing live attenuated FHV 1 strain G2620A (≥10^5.8^ PFU/dose), live attenuated FCV strain F9 (≥10^4.6^ PFU/dose), FPL strain MW-1 (≥10^5.2^ TCID_50_/dose), live attenuated *C. felis* strain Baker (≥10^4.1^ TCID_50_/dose) and an RNA particle expressing the envelope glycoprotein of FeLV (≥10^6.6^ RP/dose). The titers used for the laboratory efficacy studies were the minimum protective doses for all components. The titers used for the field trial were 10^6.8^ PFU/dose (FHV), 10^7.0^ PFU/dose (FCV), 10^5.8^ TCID_50_/dose (FPL), 10^5.9^ TCID_50_/dose (*C. felis*) and 10^7.1^ RP/dose (RP-FeLV). Construction of the RNA particle expressing the envelope glycoprotein is described elsewhere [15]. The RNA particle expressing green fluorescent protein (RP-GFP) was constructed similar to RP-FeLV, using the GFP gene instead of the FeLV envelope glycoprotein [15]. Commercially available vaccines used in the clinical field study were Nobivac Rabies (MSD Animal Health), Purevax RCPCh, Purevax FeLV, and Rabisin (Boehringer Ingelheim, Ingelheim, Germany).

Nobivac^®^ Solvent (MSD Animal Health) is phosphate-buffered water and was used to resuspend the lyophilized MSD Animal Health vaccines. For resuspension of the Boehringer Ingelheim vaccines either Purevax FeLV or water for injections was used.

### 2.2. Efficacy Studies

#### 2.2.1. Study Designs

Onset of immunity (OOI) and duration of immunity (DOI) studies were performed for all the components of the Nobivac NXT HCPChFeLV combination vaccine to determine efficacy. All studies were performed in healthy, specified pathogen-free (SPF) European short-haired cats. Inclusion involved a veterinary clinical examination, and all animals were judged to be fit before being included in the studies. Randomization was performed according to sex and litter, but no specific blinding procedures for group allocation. The primary vaccination schedule consisted of an initial vaccination at 8 to 9 weeks of age followed by a booster vaccination 4 weeks later. All OOI studies involved challenge 1 week after the booster vaccination. The FHV, FCV, and FeLV DOI studies involved challenge one year after completion of the primary vaccination schedule (1-year DOI), or 3 years after completion of the primary vaccination schedule and administration of a booster at 1 year (3-year DOI).

**Figure 1:**
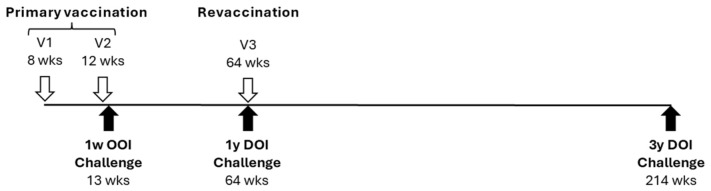
Design of efficacy studies. V1: first vaccination at 8 weeks of age. V2: booster vaccination 4 weeks after first vaccination. V3: revaccination, 1 year after V2.

For FPL, a DOI of 3 years was determined after completion of the primary vaccination schedule and without revaccination at 1 year. For *C. felis*, a DOI of 1 year after completion of the primary vaccination series was conducted (see Figure 1 and Table 1).

#### 2.2.2. Vaccination Procedures

All vaccines were administered subcutaneously. Inoculation of horses with the RP-GFP construct was performed subcutaneously and intramuscularly.

#### 2.2.3. Challenge Procedures

For FHV and FCV efficacy studies at all time points, all cats were challenged intranasally with virulent FHV-1 or virulent FCV, in line with Ph. Eur. Monographs 1102E and 1206, respectively [18]. The clinical parameters of disease assessed post challenge included clinical signs, body temperature and body weight. The excretion of challenge virus was measured post challenge.

For the OOI and 1-year DOI FPL efficacy studies, cats were challenged intraperitoneally (i.p.) with FPL virus, in line with Ph. Eur. Monograph 0251 [18]. Post challenge, the cats were monitored for clinical signs of disease, weight loss, pyrexia, and leukopenia. The excretion of challenge virus in the feces was measured after challenge. Three-year DOI efficacy was based on serological responses, in which a virus-neutralizing titer higher than 1:32 was considered protective.

For FeLV challenge procedures in all efficacy studies, cats were inoculated twice, two days apart, via the intraperitoneal route, with FeLV strain 61E, in line with Ph. Eur. Monograph 1321 [18]. No immunosuppressants were used in preparation for or during challenge procedures. As clinical signs are rarely seen in acute FeLV infections, FeLV antigenemia was evaluated from 3 to 15 weeks post challenge to determine if cats were persistently infected with FeLV. In line with Ph. Eur monograph 1321, persistent antigenemia was defined as the presence of FeLV (p27 antigen) detected in the serum, for three consecutive weeks or on five occasions, consecutive or not, between the third and fifteenth week post-challenge.

In *C. felis* efficacy studies, all cats were challenged via the intraocular and intranasal routes with *C. felis*, strain Cello, in line with Ph. Eur. Monograph 2324 [18]. Post challenge, the cats were monitored for clinical signs of disease and pyrexia. The excretion of *C. felis* post challenge was assessed by quantitative re-isolation of nasal and conjunctival swab samples.

The serological antibody responses to FHV, FCV, and FPL were measured throughout the studies. Rabies-specific antibody responses were measured throughout the duration of immunity studies.

### 2.3. Serological Analyses

#### 2.3.1. FHV- and FCV-Neutralizing Antibodies

FHV- or FCV-neutralizing serum antibody titers were determined by making serial serum dilutions and incubating them with an equal volume of a known titer suspension of FHV or FCV, respectively. These dilutions were inoculated into wells containing CRFK cells [19] in micro-titration plates. After an incubation period of 5 days at 37 °C in a humidified atmosphere, the monolayers were examined for the presence of FHV or FCV, respectively. The virus-neutralizing titer is expressed as the reciprocal of the highest dilution of the serum that shows complete neutralization of the virus. The VN titer of the serum is measured by determining VN_50_ with the Spearman–Kärber method.

#### 2.3.2. FPL-Neutralizing Antibodies

FPL-neutralizing serum antibody titers were determined by making serial serum dilutions and incubating them with an equal volume of a known titer suspension of FPL. These dilutions were inoculated into wells containing CRFK cells in micro-titration plates. After an incubation period of 4 days at 37 °C in a humidified atmosphere, the monolayers were examined for the presence of virus by immunofluorescence using an FPL-specific monoclonal and a conjugated secondary antibody. The virus-neutralizing titer is expressed as the reciprocal of the highest dilution of the serum that shows complete neutralization of the virus. The VN titer of the serum is measured by determining VN_50_ with the Spearman–Kärber method.

#### 2.3.3. *C. felis*-Specific Antibody Responses

*C. felis*-specific serum antibody titers were determined by making serial serum dilutions in microtiter plates containing McCoy cells infected with *C. felis*. *C. felis*-specific antibodies were detected by incubation with a conjugated anti-feline IgG monoclonal antibody. Specific fluorescence indicates the presence of *C. felis* antibodies in the serum. The *C. felis*-specific antibody titer is the serum dilution at which 50% of the wells show specific fluorescence of *C. felis* inclusions, calculated according to the Spearman–Kärber method.

#### 2.3.4. FeLV-Neutralizing Antibodies

FeLV-neutralizing serum antibody titers were determined by making serial serum dilutions and incubating them with an equal volume of a known titer suspension of FeLV. These dilutions were inoculated into wells containing CRFK cells in micro-titration plates. After an incubation period of 6 days at 37 °C in a humidified atmosphere, the monolayers were examined for the presence of virus by immunofluorescence using an FeLV-specific monoclonal antibody. The absence of specific fluorescence indicates virus neutralization. The VN titer is the serum/plasma dilution at which all wells show complete neutralization.

#### 2.3.5. Rabies-Neutralizing Antibodies

Antibodies for rabies were determined by a Rapid Fluorescent Focus Inhibition Test (RFFIT) according to OIE Rabies Chapter 3.1.17 [20]. The RFFIT measures the ability of serum rabies antibodies to neutralize the infection of cells in culture.

### 2.4. Reisolation Procedures

#### 2.4.1. FHV and FCV

Re-isolation of FHV and FCV from oral swabs was performed on CRFK cells. The swab samples were serially diluted and inoculated on CRFK cells in micro-titration plates and incubated for 5 days at 37 °C in a humidified atmosphere. After incubation, the plates were read out on CPE and titers were calculated in accordance with the method of Spearman–Kärber and were expressed in log_10_ TCID_50_/mL.

#### 2.4.2. FPL

Re-isolation of FPL from rectal swabs was performed on CRFK cells. The swab samples were inoculated on CRFK cells in micro-titration plates and incubated for 4 days at 37 °C in a humidified atmosphere. After incubation, the cells were fixed and FPL was detected by immunofluorescence using an FPL-specific Moab and a conjugated secondary antibody. The wells were examined for FPL-specific fluorescence and titers were calculated in accordance with the method of Spearman–Kärber and were expressed in log_10_ TCID_50_/mL.

#### 2.4.3. *C. felis*

Re-isolation of *C. felis* from conjunctival and nasal swabs was performed on L929 cells. The swab samples were serially diluted and inoculated on L929 cells in micro-titer plates. The plates were then centrifuged at 1600× *g* for 1 h at 37 °C. Next, the medium was removed, and fresh medium was added to each well. The plates were incubated for 6 days at 37 °C in a humidified atmosphere. The monolayers were examined for the presence of *C. felis* by immunofluorescence using an anti-*C. felis* monoclonal and a conjugated secondary antibody. The titers were calculated in accordance with the method of Spearman–Kärber and were expressed in log_10_ TCID_50_/mL.

#### 2.4.4. RP-GFP

Re-isolation of RP-GFP from rectal swabs, nasal swabs, homogenized organ samples, spinal fluid samples and serum samples was performed on Vero [21] cells. The samples were inoculated on Vero cells in micro-titration plates and incubated overnight at 37 °C in a humidified atmosphere. After incubation, the wells were examined for RP-GFP by specific fluorescence.

### 2.5. Safety Studies

Studies in cats were performed to assess safety of a single and repeated dose (*n* = 11 vaccinates, *n* = 5 controls), two studies into safety of an overdose of viral components (*n* = 10 vaccinates and *n* = 11 vaccinates, *n* = 5 controls, respectively) and a specific FPL overdose safety study (*n* = 5). Studies used specific-pathogen-free kittens of 8 to 9 weeks of age. Nobivac NXT HCPChFeLV vaccines were administered subcutaneously, and Nobivac Rabies was administered subcutaneously, concurrently on the flank, in selected studies (on the same day but not mixed). Monitoring included daily clinical observations, local injection site assessments, body temperature measurements, serology for vaccine components and rabies, periodic leucocyte counts, and swab sampling for re-isolation of vaccine components.

As a model to assess the safety of the RNA particle construct, dissemination, shed and spread of model construct RP-GFP was studied in horses. Horses were selected as they are the natural host of VEEV on which the RP platform is based. Thirteen Shetland ponies were divided into three groups. On day 0, ponies in group 1 (*n* = 9) were inoculated intramuscularly and subcutaneously with RP-GFP (dose: 10^9^ RNA particles). Animals in group 2 (*n* = 2) served as sentinels and were housed together with animals from group 1. Animals in group 3 (*n* = 2) served as negative controls for the laboratory tests and were housed separately from the other groups. Scheduled necropsies on horses in group 1 were carried on specific days until day 7, and on days 14 and 16 on horses of groups 2 and 3, respectively (end of study). During necropsies, organs and tissues were collected for re-isolation purposes. Clinical observations were performed and body temperatures were measured daily for all horses of groups 1 and 2. Blood samples were taken for serological analyses. Nasal and rectal swabs were taken daily in groups 1 and 2 and from horses in group 3 at end of the study for re-isolation purposes.

### 2.6. Clinical Field Study

A clinical field study was conducted to demonstrate the safety and efficacy of Nobivac NXT HCPChFeLV in concurrent non-mixed use with Nobivac Rabies in cats under field conditions compared to a positive control group. In total, 71 healthy kittens (8 weeks old) and 71 healthy adult cats (at least one year old) of various (mixed) breeds were included and allocated randomly to either test or control group. The test groups were vaccinated with Nobivac NXT HCPChFeLV on day 0 and again three to four weeks later. The test groups were also vaccinated with Nobivac rabies at the second vaccination. The control groups were vaccinated with Purevax RCPCh and Purevax FeLV on day 0 and again three to four weeks later. The control groups were also vaccinated with Rabisin at the second vaccination.

The safety was assessed based on daily observations of injection sites and systemic reactions from 3 days before until 14 days after each vaccination. Rectal temperatures were measured daily in all animals from three days before until 4 days after each vaccination (including 4 h after vaccination). Blood samples were taken from all animals in the study prior to both vaccinations and two weeks after the last vaccination to assess antibody responses to FHV, FCV, FPL, *C. felis*, and FeLV.

### 2.7. Statistical Methods

Total clinical scores recorded post challenge of FHV, FCV, and *C. felis* efficacy studies were analyzed by means of Generalized Estimating Equations using a cumulative logit model, accounting for the correlation in the repeated measurements of an animal. The response variable was included as an ordinal response in the GEE model using a multinomial distribution with cumulative logit as the link function. For analysis of body weight ratios, the body weights recorded on the day of challenge were set at 100% for each individual animal and the body weights recorded each day after challenge were expressed as % of the body weight on the day of challenge. Statistical analysis was performed by ANOVA with repeated measurements. Survival rates in the FHV efficacy studies were analyzed using Fisher’s exact test. For statistical analysis of FeLV antigenemia, Fisher’s exact test was conducted to compare the proportions. Subsequently, the prevented fraction (PF) of FeLV persistent antigenemia and its 95% confidence interval was calculated. Viral and bacterial excretion, represented by titers determined in swab materials obtained after challenge, were analyzed by ANOVA with repeated measurements.

## 3. Results

### 3.1. Efficacy Studies

#### 3.1.1. FHV

**Figure 2:**
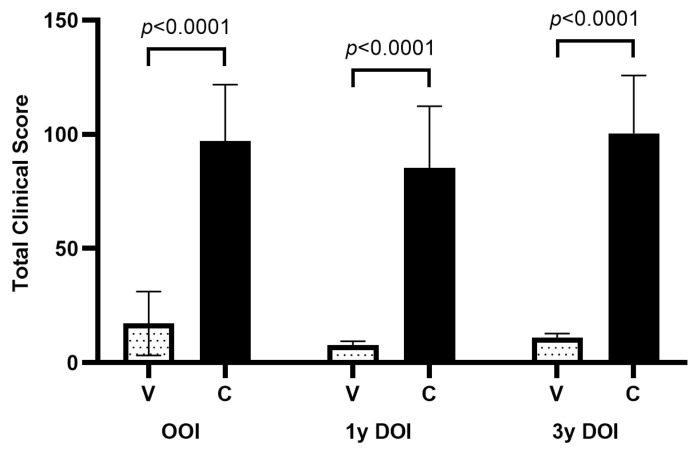
Mean total clinical scores after challenge with FHV in vaccinates (V) and controls (C) at OOI, 1-year DOI, 3-year DOI.

Across the OOI, 1-year DOI and 3-year DOI studies, vaccinated cats developed only mild to moderate, transient upper-respiratory signs after FHV challenge. Most commonly observed clinical signs were sneezing, lachrymation and mild nasal discharge beginning around day 4 or 5 and resolving by day 14. Total clinical scores in vaccinate groups were low, with median values of 4 in the OOI, 2 in the 1-year DOI, and 15 in the 3-year DOI. Vaccinates showed brief pyrexic spikes around days 4 and 5 p.c. that usually resolved within 1 to 3 days, with only a small subset experiencing somewhat prolonged or more pronounced signs. By contrast, unvaccinated controls developed earlier, more frequent and more severe disease starting from day 3 or 4, with multiple concurrent clinical manifestations (respiratory, ocular, systemic and sometimes neurologic signs), and significantly higher and total clinical scores compared to vaccinated groups (median values of 105 in the OOI, 53 in the 1-year DOI, and 51 in the 3-year DOI), and more prolonged periods of pyrexia. Vaccinates had significantly lower total clinical scores than controls in every study (see Figure 2).

**Figure 3:**
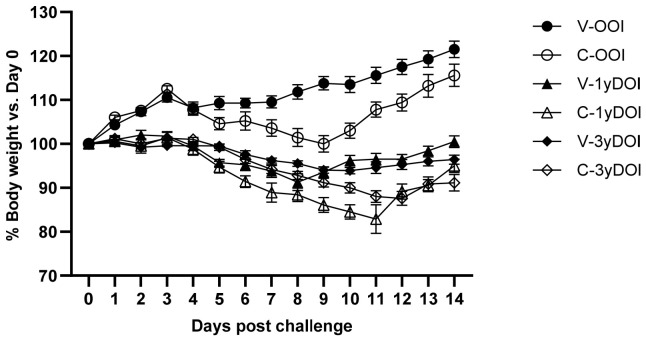
Percent body weight reduction from baseline after FHV challenge in vaccinates (V) versus controls (C) at OOI, 1-year DOI, and 3-year DOI.

**Figure 4:**
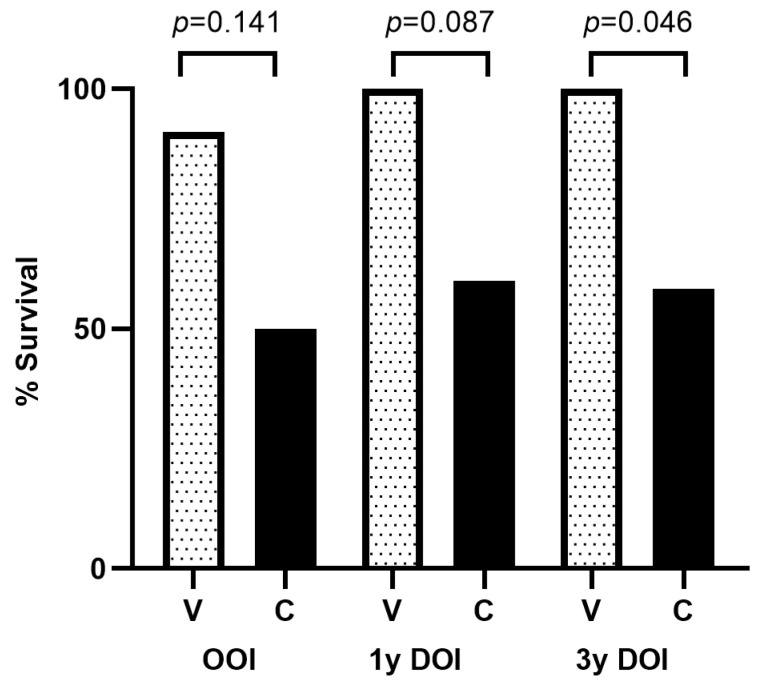
Percent survival after challenge with FHV in vaccinates (V) versus controls (C) at OOI, 1-year DOI, and 3-year DOI.

In the OOI study, the body weight development of the kittens in the unvaccinated control group was shown to be significantly reduced compared to the kittens in the vaccinated group (*p* = 0.0016). Furthermore, in both the 1-year and 3-year DOI studies, vaccination was shown to significantly reduce body weight loss (*p* = 0.0003 and 0.0067, respectively) (Figure 3). Mortality was markedly reduced by vaccination, with survival rates of 90% vs. 50% in the OOI study, and 100% vs. 60% and 58% in the 1-year and 3-year DOI studies, respectively (Figure 4).

#### 3.1.2. FCV

**Figure 5:**
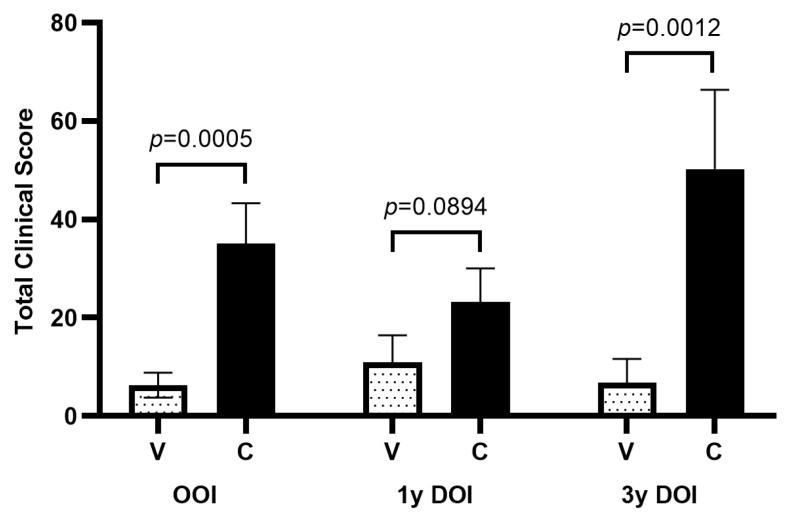
Mean total clinical scores post-challenge with FCV in vaccinates (V) versus controls (C) at OOI, 1-year DOI, and 3-year DOI.

Across the OOI, 1-year DOI and 3-year DOI FCV challenge studies, vaccinated cats predominantly developed small, short-lived oral and nasal ulcers or remained asymptomatic. Brief periods of pyrexia that were resolved within 1 to 3 days were noted. Total clinical scores in vaccinates were low, with median values of 4 in the OOI study, 5 in the 1-year DOI study, and 1 in the 3-year DOI study. A substantial fraction of vaccinates in each arm showed no signs by day 14. By contrast, unvaccinated controls developed earlier, more frequent and more severe disease with larger, more numerous and longer-lasting ulcers and accompanying systemic signs (poor condition, ocular and/or nasal discharge, occasional dyspnea, dehydration, excessive salivation and in some cases neurological signs or lameness). Sustained weight loss was seen in several animals (including occasional losses > 10%), and more prolonged periods of pyrexia compared to vaccinates (Figure 5).

**Figure 6:**
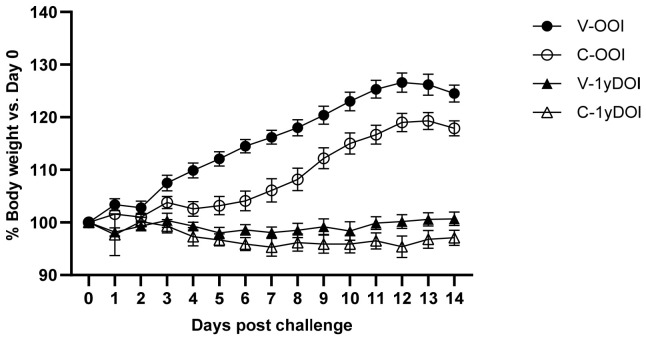
Percent body weight reduction from baseline after FCV challenge in vaccinates (V) versus controls (C) at OOI and 1-year DOI.

In the OOI study, the body weight increase in the kittens in the unvaccinated control group was shown to be significantly reduced compared to the kittens in the vaccinated group (*p* = 0.0011). In the 1-year DOI study, the protective effect on body weight impact due to FCV infection was trending though not statistically significant (*p* = 0.1303). One of the controls in the 3-year DOI required humane endpoint euthanasia. Total clinical scores were significantly higher compared to vaccinated groups in the OOI and 3-year DOI studies, with median values of 36 (*p* = 0.0005) and 38 (*p* = 0.0012), respectively, and trending in the 1-year DOI (15, *p* = 0.0894) (Figure 6).

#### 3.1.3. FPL

**Figure 7:**
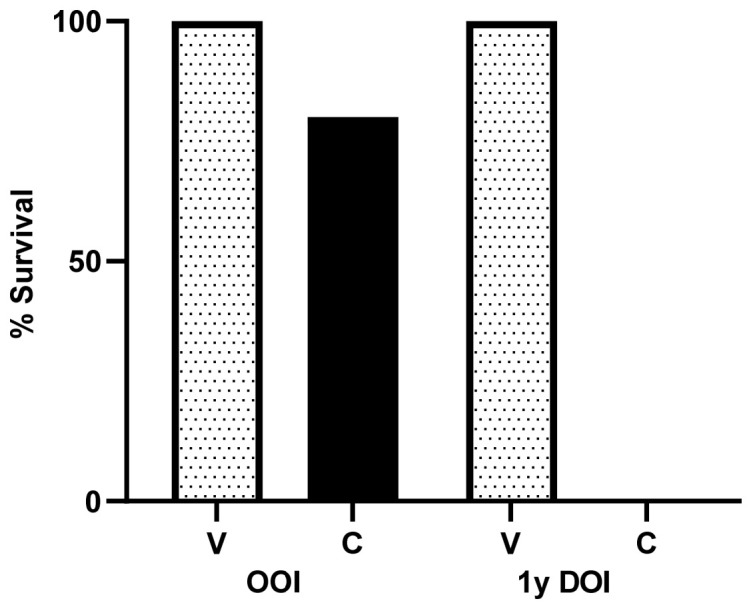
Percent survival after challenge with FPL in vaccinates (V) versus controls (C) at OOI and 1-year DOI.

Post challenge, the vaccinated cats did not develop any clinical signs of disease. All vaccinated cats remained within normal body temperature limits post challenge, apart from one vaccinate in the 1-year DOI study, which was observed to have pyrexia on day two post challenge that resolved by the following day. In contrast, in both OOI and 1-year DOI studies, unvaccinated control cats developed marked clinical disease after challenge. In the OOI study most controls remained clinically normal except for one cat that developed malaise, depression, reduced appetite with sustained weight loss, dehydration, diarrhea, pyrexia and leukopenia on day 6 and was euthanized on welfare grounds. All control cats showed early weight loss (70–110 g) on day 1 post-challenge, with five of six exhibiting further loss by day 7. A spike in pyrexia was also observed in the unvaccinated control cats between 1 and 3 days post challenge. In the 1-year DOI study all control cats showed clinical signs of FPL from days 5–7, including malaise, depression, anorexia, dehydration, poor condition and bile vomiting. All cats showed progressive weight loss, and one cat had pyrexia on day 6. Because of the severe clinical disease and progressive weight loss, all control cats were euthanized on humane grounds on day 6 or 7 (Figure 7).

**Figure 8:**
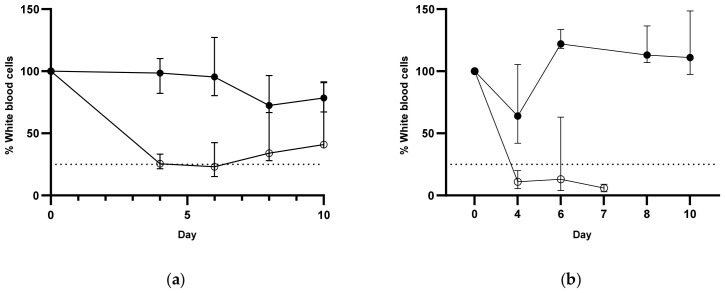
White blood cell counts as percent of baseline median after challenge with FPL in vaccinates (●) versus controls (○) at (**a**) OOI; (**b**) 1-year DOI. Dotted line indicates 75% reduction versus baseline.

In both OOI and 1-year DOI studies the unvaccinated controls developed a rapid and marked leukopenia from day 4 post-challenge, whereas vaccinated cats showed little to no drop in white blood cell (WBC) counts. In the OOI study, four of six control cats were leukopenic by day 6; two others did not meet the 75% reduction threshold but still fell substantially to 29% and 34% of their baseline counts, and one control reached 1% of its baseline count on day 6. This is the same cat that showed clinical signs of disease and was euthanized. In the 1-year DOI study, one vaccinated cat showed a transient drop in WBC counts leukopenia on day 4 (25%) that fully resolved by day 6 (138%) and was not associated with clinical disease. In contrast, all five unvaccinated controls became leukopenic from day 4 or 6, reaching the 75% reduction criterion with WBC counts at 1 to 12% of their baseline, the timing of which preceded the development of clinical signs one day later. One control WBC rebounded by day 6, but the remaining animals remained below 25% and as all animals were euthanized on day 6 or 7, no later analyses were performed (Figure 8).

#### 3.1.4. FeLV

Following challenge in the OOI study, mild transient clinical signs were observed in one unvaccinated cat 4 weeks post infection. This cat presented with transient pyrexia, loss of appetite and mild hyperpnea for one day. No clinical signs of disease were observed in other cats throughout the studies.

**Figure 9:**
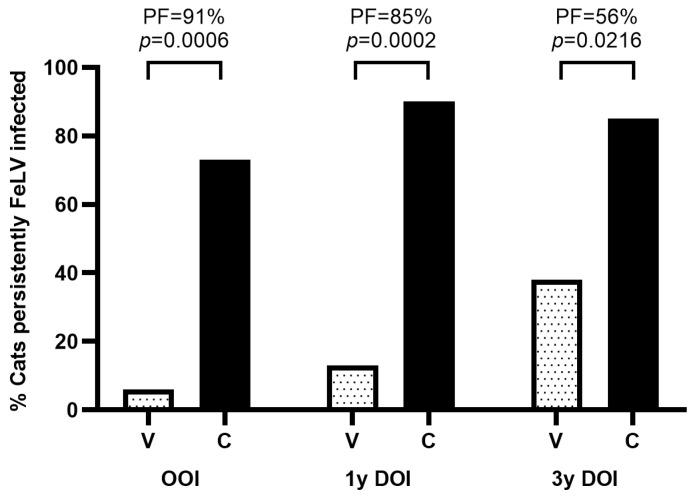
Percent of vaccinated (V) and control (C) cats persistently FeLV antigenemic at OOI, 1-year DOI, and 3-year DOI.

In all efficacy studies, vaccinates were shown to be significantly protected from a robust FeLV challenge infection compared to vaccinates. In the OOI study, 72.7% (8/11) of unvaccinated cats were persistently antigenemic, versus just 6.3% (1/16) in the vaccinated group. The prevented fraction was 91% and statistically significant (*p* = 0.0006). In the 1-year DOI study, 90.0% (9/10) of controls were persistently antigenemic versus 13.3% (2/15) of vaccinates, resulting in a preventive fraction of 85% (*p* = 0.0002). In the 3-year DOI study, 84.6% (11/13) of controls were persistently antigenemic versus 37.5% (6/16) of vaccinates, resulting in a preventive fraction of 56% (*p* = 0.0216) (Figure 9).

#### 3.1.5. *C. felis*

**Figure 10:**
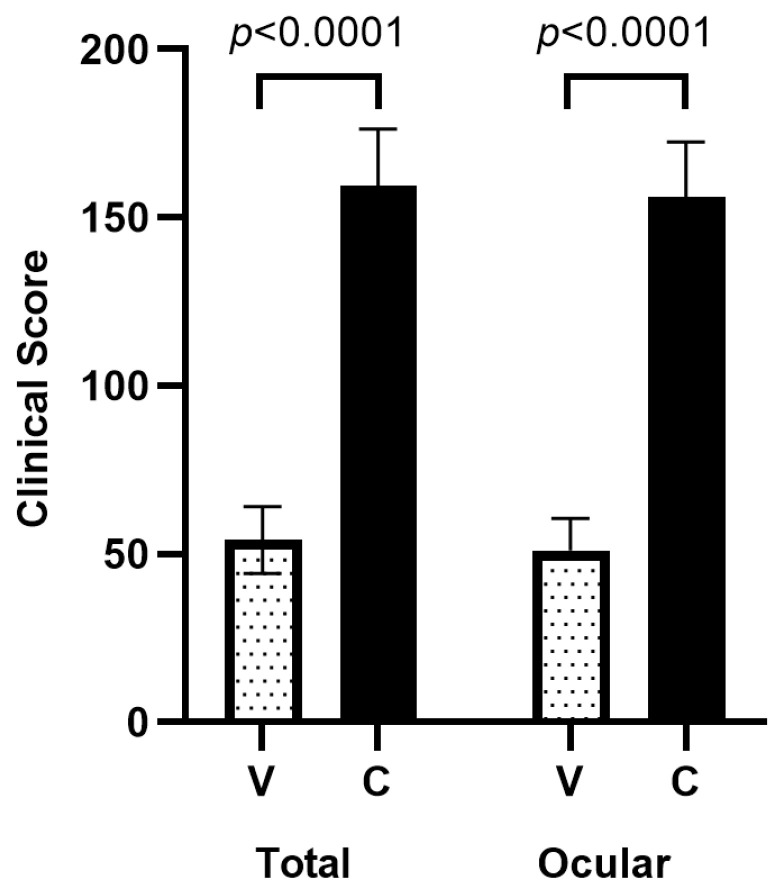
Mean total clinical and ocular scores post challenge with *C. felis* in vaccinates versus controls.

In both OOI and 1-year DOI studies, vaccinated cats developed predominantly mild, mainly ocular clinical signs of disease, such as mild to marked conjunctivitis, mucopurulent discharge with occasional lachrymation, and occasional transient general health or nasal signs in a minority of animals, from days 3 to 6 after *C. felis* challenge onward. No pyrexia was observed in the OOI study and transient pyrexia was seen in six out of ten vaccinates in the 1-year DOI study, usually resolving in a single day, and one cat had intermittent mild pyrexia later in the study. In contrast, unvaccinated controls exhibited earlier onset, more frequent and more severe disease. In the OOI study, controls developed signs by day 4 and by day 6 nine of ten showed persistent clinical signs of *C. felis* disease including mucopurulent discharge, conjunctivitis, lachrymation and some nasal discharge. The control cats had statistically significant higher total clinical scores (*p* < 0.0001) and ocular scores (*p* < 0.0001), compared to vaccinates (Figure 10). The controls in the 1-year DOI study showed significantly more general-health impairment (*p* = 0.0007), higher ocular clinical scores in the first two weeks p.c. (*p* = 0.0202) and more mucopurulent discharge.

### 3.2. Excretion of Challenge Strains

#### 3.2.1. FHV

Shedding in both vaccinates and controls started two or three days post challenge. The excreted FHV titers were found to be similar in both groups up to 7 days post challenge, after which the vaccinated groups reduced viral excretion to significantly lower levels than control groups, with the majority of cats testing negative in the final 4 to 5 days of the 14 day monitoring period. Overall, the vaccinated groups were shown to have significantly lower levels of viral excretion compared to the control cats in all studies (OOI *p* < 0.0001; 1-year DOI *p* < 0.0001; 3-year DOI *p* = 0.0028).

#### 3.2.2. FCV

Shedding in both vaccinates and controls started one or two days post challenge and persisted until 14 days post challenge. In general, up until 4 to 6 days after challenge the FCV titers between group 1 and group 2 were found to be similar, after which vaccinated groups showed reduced virus excretion compared to control groups. Over the 14-day monitoring period, the vaccinated groups were shown to have highly significantly lower levels of viral excretion compared to the control cats in all studies (OOI *p* < 0.0001; 1-year DOI *p* = 0.0044; 3-year DOI *p*< 0.0001).

#### 3.2.3. FPL

**Figure 11:**
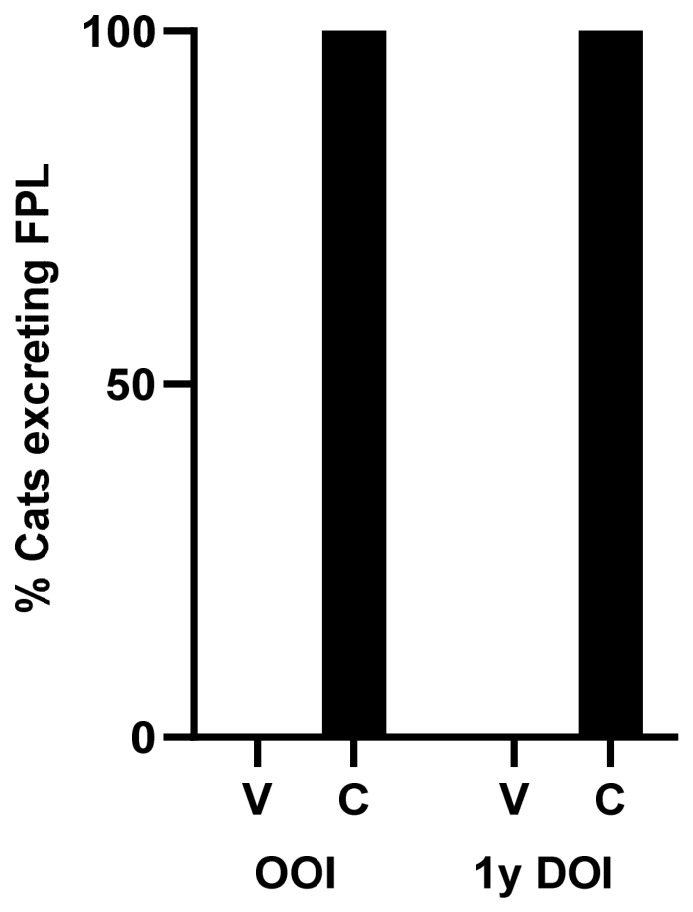
Percent of cats excreting FPL post challenge in vaccinates (V) versus controls (C) at OOI and 1-year DOI.

Vaccinates in both studies did not shed FPL throughout the 14-day monitoring period. By contrast, all controls tested positive from 1 or 2 days post challenge and excreted virus for 4 to 8 days or until euthanasia due to humane endpoints (Figure 11).

#### 3.2.4. *C. felis*

*C. felis* excretion was assessed by quantitative re-isolation from nasal and conjunctival swabs collected daily after challenge in both OOI and 1-year DOI studies. In both studies, the vaccinated cats were shown to excrete *C. felis* to significantly lower levels compared to controls in both nasal as well as conjunctival swab samples (OOI *p* < 0.0001 and *p* = 0.0007; DOI *p* = 0.0004 and *p* = 0.0022 for nasal and conjunctival swabs, respectively) over the four-week monitoring period.

### 3.3. Serological Responses

#### FHV, FCV, FPL, and Rabies Virus Neutralizing Antibodies

Serological responses against the FHV, FCV, and FPL components were analyzed during all OOI, 1-year and 3-year DOI efficacy studies. Serology for FeLV was not performed. All cats were shown to be seronegative at the start of the studies.

**Figure 12:**
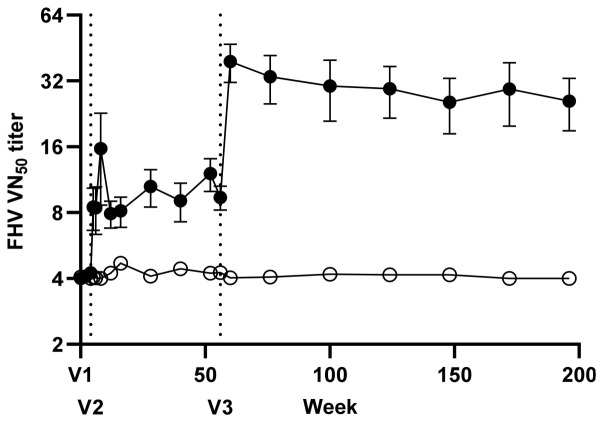
FHV-neutralizing titers post primary vaccination and 1 year booster for 4 years in vaccinates (●) versus controls (○). V1 and V2 (dotted line): primary vaccination. V3 (dotted line): revaccination.

**Figure 13:**
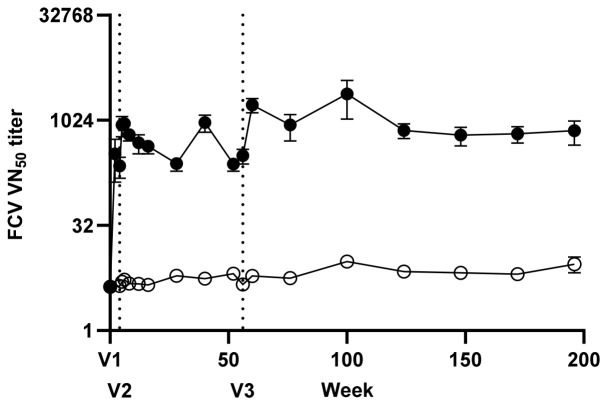
FCV-neutralizing titers post primary vaccination and 1 year booster for 4 years in vaccinates (●) versus controls (○).V1 and V2 (dotted line): primary vaccination. V3 (dotted line): revaccination.

Figure 12 and Figure 13 show the results of FHV and FCV VN antibody titers obtained in the 3-year DOI studies for the full 4-year study period, in which cats were vaccinated at 8 to 9 and 12 to 13 weeks of age (primary vaccination) and re-vaccinated 1 year after primary vaccination. FHV VN titers were slow to develop, a common feature of this pathogen. Nevertheless, FHV VN antibody titers were boosted by the second vaccination and especially after re-vaccination one year after primary vaccination, with titers increasing three to four-fold and enduring over the three years. FCV VN antibodies developed quickly after the first vaccination and in general a four-fold increase was seen after the second vaccination of the primary vaccination scheme. Re-vaccination after one year again boosted titers that were then maintained over the three years.

**Figure 14:**
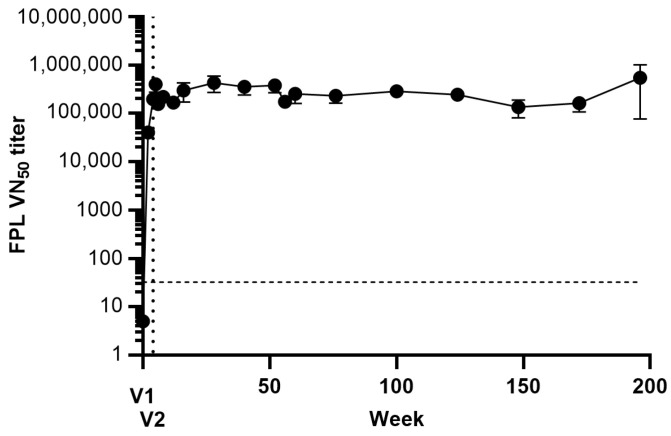
FPL-neutralizing titers post primary vaccination for 3 years in vaccinates. Dotted line indicates 1:32 protective VN titer.

Figure 14 shows the results of FPL VN antibody responses obtained over 3 years after completion of just the primary vaccination schedule. FPL VN antibody titers were induced quickly, with all cats showing titers well above the 1:32 minimum protective titer after a single vaccination. High titers were maintained for 3 years after primary vaccination.

**Figure 15:**
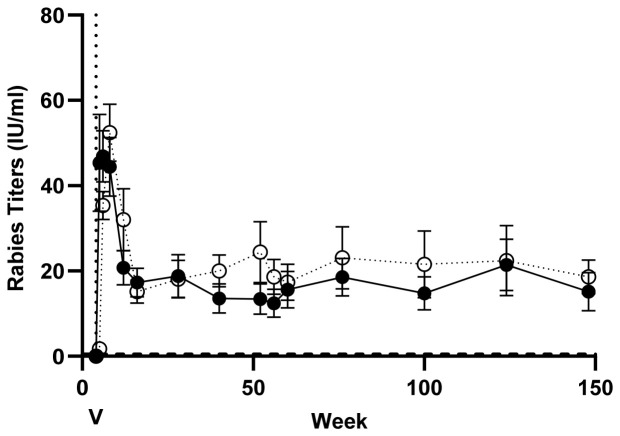
Rabies virus-neutralizing titers post concurrent vaccination with Nobivac Rabies for 3 years in vaccinates (●) versus controls (○). Dashed line indicates minimum protective titer (0.5 IU/mL).

Figure 15 shows the results of rabies virus-neutralizing antibody responses obtained over 3 years after concurrent vaccination with Nobivac Rabies at the second vaccination moment of the primary vaccination schedule. Unvaccinated control cats were also vaccinated with Nobivac Rabies at this time point. Rabies virus-neutralizing antibody titers were induced quickly, with all cats seroconverting one week after vaccination. High titers well above the 0.5 IU/mL minimum protective titer were maintained for 3 years after primary vaccination.

### 3.4. Safety in Cats

The safety profile of Nobivac NXT HCPChFeLV was assessed in laboratory safety studies conducted in young SPF kittens. No serious adverse events were observed in any of the studies. Pyrexia occurred most often after the first vaccination and was more consistent following overdose administrations, resolving within two days in all studies. Local injection site reactions were the most frequent local reaction observed. These were small and short lived after maximum dosing and more frequent and of greater size and duration after administration of an overdose, generally diffuse, not warm and not painful, occasionally hard and not painful or soft and painful on the day of injection and without chronic progression or need for intervention.

### 3.5. RNA Particle Safety

Seroconversion to RP GFP was observed in inoculated horses from day 5 onward, whereas sentinel animals remained seronegative throughout the study. RP GFP was reisolated only once from one of the injection sites on day 1 post inoculation and was not recovered from any other tissues, swabs or sera. Furthermore, no cytopathic effect was observed in culture re-isolation procedures of other samples. These findings show that the RP vector did not disseminate, shed or spread to cohoused sentinel horses in this sensitive natural host model.

In the RP FeLV shed-and-spread studies conducted in cats, RP FeLV was never isolated from oral, nasal, conjunctival or rectal swabs in vaccinated cats in either the commercial- or maximum-dose studies. In addition, no RP FeLV was detected in sentinel cats. These findings show that RP FeLV is not shed or spread by vaccinated cats.

### 3.6. Clinical Field Trial

Vaccination with Nobivac NXT HCPChFeLV was well tolerated in both adult cats and kittens. No injection site reactions were observed in any animal from the test group following either the first or second vaccination. No adult cats in the test group exhibited abnormal general health scores after vaccination. One kitten in the test group experienced a mild adverse event consisting of reduced activity for one day, occurring 10 days after the second vaccination. Rectal temperatures did not show clinically relevant changes following either vaccination in either adult cats or kittens.

Vaccination with Nobivac NXT HCPChFeLV stimulated serological responses to all components in both adult cats and kittens, with the exception of the FeLV component. In adult cats, baseline seropositivity was already high for FCV, FPL, and *C. felis* (96%, 83%, and 96%, respectively), likely reflecting prior vaccination or field exposure, and increased to 100% for all three antigens two weeks after the second vaccination; FHV seropositivity rose from 33% before vaccination to 67% after the second dose. In kittens, pre-vaccination seropositivity for FCV, FPL, and *C. felis* was also substantial (70%, 64%, and 52%, respectively), likely due to maternally derived antibodies (MDA) or early field infection, and increased to 97 to 100% two weeks after the second vaccination, while FHV seropositivity increased from 15% at baseline to 61% following completion of the vaccination course.

## 4. Discussion

Vaccine-preventable feline infectious diseases is still a major concern amongst cat populations worldwide [10,11,12,13]. This study demonstrates that vaccination with a novel vaccine combining modified live/avirulent live and RNA particle technology (Nobivac NXT HCPChFeLV) yields substantial clinical and survival benefits against common and significant feline pathogens. Vaccinated cats showed a significant reduction in clinical signs at all post-challenge time points, including mitigation of weight loss and oral ulceration. The reduction in weight loss is of particular clinical relevance because these diseases disproportionately affect kittens, in which even modest weight loss can rapidly translate into worsened prognosis or mortality. Importantly, vaccination conferred significant protection against the characteristic leukopenia of panleukopenia; preservation of white blood cell counts has direct implications for survival and recovery in clinically affected animals and supports the vaccine’s role in altering disease severity rather than merely modifying clinical expression.

Mortality outcomes further reinforce the vaccine’s protective profile. Vaccinated animals experienced significant reductions in mortality from panleukopenia and FHV challenge, with the latter representing a notable advance given that this vaccine is the first and only licensed product to show labeled protection against FHV mortality. These mortality benefits, combined with reductions in clinical signs and laboratory markers of disease severity, indicate that vaccine-induced immunity translates into meaningful improvements in patient-centered outcomes.

Serological data corroborate these clinical findings. In the laboratory efficacy studies, vaccination induced rapid primary serological responses, strong memory responses after revaccination, and robust anamnestic responses upon challenge. Although FHV-specific titers were low after primary vaccination (a common feature of FHV vaccines), revaccination induced high VN antibody titers that were maintained for years, indicating durable immunological memory. This pattern—waning circulating antibody titers with preserved memory responses—is consistent with long-lived cellular and memory B-cell compartments that can rapidly reconstitute protective antibody levels upon revaccination or exposure to infection. In the field trial all adult cats seroconverted post vaccination, kittens demonstrated high level of pre-existing antibodies against FCV, FPL, and *C. felis*, indicating presence of MDA or prior exposure. Regardless, 97 to 100% of these kittens seroconverted after completion of primary vaccination, indicating that the vaccine was able to overcome MDA. For FHV, only 15% of the kittens had VN antibodies, increasing to 61% after vaccination. For alphaherpesviruses in general, cellular immunity is thought to be a better biomarker for immune status than (virus-neutralizing) antibodies [22], which may be corroborated by the protective effect of vaccination against FHV despite low levels of VN antibodies in the laboratory efficacy studies. From a practical standpoint, these data support the vaccine’s capacity to provide long-term protection even when measurable titers fall below arbitrary thresholds, provided that memory responses remain intact. This also demonstrates the ability of the vaccine to function under real-world conditions such as prior exposure, prior vaccination, and MDA interference.

At the population level, reductions in viral and bacterial shedding for all tested antigens are particularly important. Lower excretion decreases transmission potential and consequently contributes to herd immunity, an effect that is critical in multi-cat environments such as shelters, catteries, and household settings with multiple cats and/or mixed indoor–outdoor populations [23,24]. The observed decrease in shedding therefore amplifies the individual-level protection by lowering community exposure risks and helping to prevent outbreaks.

Safety assessments in both controlled laboratory and field settings support a favorable tolerability profile. Laboratory safety studies documented a low rate of post-vaccination reactions, generally limited to mild swelling consistent with an expected immune response. Importantly, field safety evaluation of 150 administered doses identified no injection-site reactions, fevers, or clinically relevant health changes, indicating that the product is well tolerated in real-world use, including in kittens.

This study also demonstrated high efficacy, safety, and no shed and spread for a novel RNA Particle vaccine containing the FeLV envelope glycoprotein. The preventive fractions in both 1 week onset and 1 year duration of immunity studies were well above the 75% requirement as per the Ph. Eur. Monograph for FeLV vaccines (91% and 85%, respectively). Although statistically significant, the preventive fraction decreased to 56% in the 3 year duration of immunity study, indicating that beyond 1 year after primary vaccination, a reduction rather than a prevention of antigenemia is induced. It should be noted that this these studies employed a robust challenge model that did not include the use of immunosuppressants—cats received two intraperitoneal inoculations two days apart with FeLV strain 61E—yet still demonstrated substantial vaccine efficacy, as reflected in preventive fraction estimates and p27 antigenemia. Such stringent challenge conditions likely represent a conservative test of protective capacity and strengthen confidence that observed effects are biologically meaningful in the face of high infectious pressure. Under natural conditions, exposure typically occurs via saliva, either transiently or during prolonged co-mingling, and can occur also through bites and vertically from queen to kitten. The experimental challenge model was therefore severe, but the vaccine still demonstrated significant protection against a highly virulent challenge. These results allow an informed decision as to how to deploy revaccination strategies, using a risk-based evaluation of individual animals. Yearly revaccination would afford prevention of persistent viremia, particularly for cats at higher risk of exposure, whereas revaccination every 2 to 3 years could be considered for cats at a lower risk of FeLV exposure.

While veterinary benchmarking against other commercially available FeLV vaccines should be approached cautiously, relevant efficacy data have been published elsewhere. These include publications on inactivated and recombinant viral vector vaccines that were compared in vaccination/challenge studies using the same FeLV challenge strain as used in the efficacy studies reported here, FeLV subtype A strain 61E [15,25,26]. These studies reported efficacy results three weeks to three months after completion of primary vaccination schedules consisting of two vaccinations, resulting in preventive fractions ranging from 50% to 93% for Purevax FeLV, 93% to 100% for Nobivac Feline 2-FeLV, 80% for Versifel FeLV, and 100% for Nobivac NXT FeLV [15,25,26], which contains the same RNA particle vaccine as reported here [15]. As reported here, the efficacy profile of the RNA particle vaccine component for FeLV in Nobivac NXT is at least equivalent to current commercially available vaccines. Furthermore, it provides a unique one week onset of immunity and a low application volume of 0.5 mL. Finally, no replication-competent FeLV was shed from vaccinated cats or from vaccinated horses in VEEV-related testing, even at the maximum dose and over a 14-day observation period, addressing biosafety concerns about vaccine-derived shedding.

Several limitations warrant consideration. Controlled challenge studies, while valuable for demonstrating biological efficacy, may not fully model real-life conditions cats encounter. These include field exposure dynamics, co-infections, or host factors such as genetic variability and concurrent disease. Except for the FPL component, for which threshold titers of virus-neutralizing antibodies have been shown to correlate with protection [27], no clear biomarkers or correlates of protection are available. Protection against infectious diseases is mediated through an intricate combination of innate, humoral and cellular immune responses, and host factors, the details of which remain to be elucidated for the other vaccine components.

Taken together, these findings indicate that Nobivac NXT HCPChFeLV not only reduces disease signs and mortality but also attenuates pathogen shedding and preserves key hematologic parameters associated with survival. This combination of individual-level clinical benefit, population-level transmission reduction, durable immunologic memory, and strong safety supports the vaccine’s use as a cornerstone of preventive feline health programs.

## 5. Conclusions

In conclusion, the accumulated evidence indicates that Nobivac NXT HCPChFeLV provides robust protection against common feline pathogens; improves clinically meaningful outcomes including survival, weight loss, clinical disease, and preservation of leukocyte counts; elicits durable immunologic memory; reduces pathogen shedding with potential herd-level benefits; and has a favorable safety profile in both laboratory and field contexts. The novel RNA particle technology enables robust protection against virulent FeLV challenge with a strong safety profile and no shed and spread. These attributes support incorporation of this vaccine into comprehensive feline preventive care strategies, with continued post-marketing surveillance and field studies recommended to further refine vaccination schedules and maximize population health benefits.

## Figures and Tables

**Table 1:** An overview of the efficacy studies performed with Nobivac NXT HCPChFeLV. Numbers of cats in vaccinated (V) and control (C) groups are given between brackets.

Component	1 Week OOI	1 Year DOI	3 Year DOI *
FCV	✓ (10V; 10C)	✓ (10V; 10C)	✓ (10V; 14C)
FHV	✓ (11V; 10C)	✓ (10V; 10C)	✓ (10V; 13C)
FPL	✓ (6V; 6C)	✓ (5V; 5C)	✓ ** (5V)
*C. felis*	✓ (11V; 10C)	✓ (10V; 10C)	n.a.
FeLV	✓ (16V; 11C)	✓ (15V; 10C)	✓ (16V; 13C)

* After revaccination at 1 year; ** after primary vaccination. ✓: performed. n.a.: not applicable.

## Data Availability

The data presented in this study are available on request from the corresponding author as they are proprietary data.
